# Exploratory biomarker analysis from a phase III study of the PI3K inhibitor, copanlisib, in combination with rituximab in patients with indolent non-Hodgkin lymphoma, a retrospective study

**DOI:** 10.1007/s12094-025-03869-2

**Published:** 2025-02-21

**Authors:** Shalini Chaturvedi, Anke Weispfenning, Tine Descamps, Sara Bellinvia, David Bauer, Rong Du, Teresa Lunt, Lidia Mongay Soler, Barrett H. Childs, Pier Luigi Zinzani

**Affiliations:** 1 Bayer HealthCare Pharmaceuticals, Inc., 100 Bayer Blvd, Whippany, NJ 07981 USA; 2 Pharmaceuticals Division, Bayer AG, Berlin, Germany; 3 Bayer Plc, Reading, UK; 4 Chrestos Concept GmbH & Co. KG, Essen, Germany; 5 Bayer HealthCare Pharmaceuticals, Inc., Beijing, China; 6 Belcan, Cincinnati, OH USA; 7 IRCCS Azienda Ospedaliero-Universitaria di Bologna, Istituto di Ematologia “Seràgnoli”, Bologna, Italy; 8 Dipartimento di Medicina Specialistica, Diagnostica e Sperimentale, Università di Bologna, Bologna, Italy

**Keywords:** Biomarkers, Indolent non-Hodgkin lymphoma, Phase III clinical trials, Copanlisib, Rituximab

## Abstract

**Purpose:**

There has been increased difficulty in developing safe and effective treatment using PI3K inhibitors in heme malignancies, despite the role of PI3K/AKT being well defined in this population. This study was an attempt to conduct exploratory biomarker analysis retrospectively from the phase III CHRONOS-3 trial with the aim to identify a sub-set of patients that could benefit from treatment.

**Patients and Methods:**

Patients with CD20-positive indolent B-cell lymphoma were randomized 2:1 to receive intravenous copanlisib plus rituximab (C + R) or placebo plus rituximab (P + R). Biomarker analyses were performed to examine potential associations between treatment outcome and phosphatase and tensin homolog (PTEN) protein expression, *EZH2* and *BCL2* mutation status via next-generation sequencing, and plasma cytokine levels.

**Results:**

PTEN presence was associated with significant improvements in progression-free survival (PFS) for C + R over P + R in patients with iNHL (*P* = 0.001) and FL (*P* = 0.012). Both the mutant and wild-type *EZH2* FL patients had equal PFS benefits when treated with copanlisib. A significant improvement in PFS was observed for patients with mutant versus wild-type *BCL2* FL in the C + R arm (*P* = 0.002). Overall survival (OS) was significantly improved for patients with iNHL and low or undetectable versus high baseline IL-2 levels in the C + R arm (*P* < 0.0001, unadjusted).

**Conclusions:**

PTEN presence, *BCL2* mutations*,* and low or undetectable baseline IL-2 levels were associated with improved patient survival following treatment with C + R, supporting a potential role for these biomarkers in guiding treatment selection for patients with indolent non-Hodgkin lymphoma.

**Supplementary Information:**

The online version contains supplementary material available at 10.1007/s12094-025-03869-2.

## Introduction

B-cell non-Hodgkin lymphoma (NHL) has an estimated global incidence of more than 500,000 cases per year and is responsible for over 250,000 deaths annually [1]. Indolent NHL (iNHL), which comprises 40% of NHL cases, contains follicular lymphoma (FL), the most common lymphoma histology subtype, followed by marginal zone (MZL), small lymphocytic (SLL), and lymphoplasmacytic/Waldenström macroglobulinemia (LPL/WM). iNHL is characterized by slow growth, long disease course, and frequent association with progressive resistance to treatment and relapse [2]. Rituximab therapy is the recommended backbone therapy for the treatment of iNHL [3].

The phosphatidylinositol 3-kinase (PI3K) pathway is involved in regulating the cell cycle and is aberrantly activated in multiple cancers, including NHL [4]. Copanlisib (Aliqopa^®^, Bayer AG, Berlin, Germany), a potent pan-PI3K inhibitor with predominant activity against the PI3K-α and PI3K-δ isoforms was developed [5]. As a result of efficacy and safety findings from CHRONOS-1 (NCT01660451), copanlisib received accelerated approval from the US Food and Drug Administration (FDA) for the treatment of patients with relapsed FL who have had at least two therapies [6, 7]. Since dysregulation of this pathway is associated with several important cellular mechanisms, there were significant side effects observed in hematologic malignancies with this class of drug. On 21 April 2022, the oncology drug advisory committee (ODAC) of the FDA discussed class-wide safety findings with PI3K inhibitors in hematological malignancies. The ODAC meeting provided a framework to re-examine drug development in heme malignancies with emphasis on dose selection with respect to safety. On 13 November 2023, Bayer announced it would also voluntarily withdraw copanlisib (Aliqopa) from the market.

CHRONOS-3 (NCT02367040), a phase III study, had met its primary endpoint, showing improved progression-free survival (PFS) with C + R compared with P + R [8], with an acceptable safety profile supporting long-term use of the treatment combination [9]. To further refine the patient population who may benefit most from treatment with C + R, we sought to explore the presence of biomarkers that may predict benefit from treatment. We examined potential associations between patient survival and the expression of phosphatase and tensin homolog (PTEN) protein, a well-established negative regulator of the PI3K-Akt pathway whose dysregulation is associated with poor prognostic cancer outcomes [10, 11]. Additionally, as mutations in *BCL2* [12] and *EZH2* [13] are common in FL patients and are associated with promoting B-cell proliferation and survival, potential associations between *BCL2* and *EZH2* mutation status and survival were also evaluated. Lastly, we retrospectively assessed associations between OS and plasma levels of various cytokines. Here, we report results from exploratory biomarker analyses from the CHRONOS-3 trial with a particular focus on FL patients.

## Methods

### Study design and participants

CHRONOS-3 was a phase III study evaluating the efficacy and safety of C + R versus P + R in adults with CD20-positive indolent B-cell lymphoma, who relapsed following the last anti-CD20 monoclonal antibody-containing therapy [8]. The primary endpoint for CHRONOS-3 was PFS, and representativeness is summarized in Table S1.

### Assessments

PTEN protein expression analysis was performed via immunohistochemistry using an anti-PTEN antibody (rabbit clone 138G6, catalog #9559; Cell Signaling Technology, Inc., Danvers, MA, USA) at Mosaic Laboratories (Lake Forest, CA, USA). Tissues were stained with Dako 3,3-diaminobenzidine chromogen (Agilent, Santa Clara, CA, USA) and PTEN protein levels were centrally evaluated and scored by Mosaic Laboratories.

*EZH2* and *BCL2* mutation status was analyzed by the TruSight Oncology 500 Assay (Illumina), extraction of FFPE tissue slides was performed with the AllPrep DNA/RNA FFPE Kit, and DNA sequencing analysis was performed by Almac Diagnostics. The NextSeq 550Dx Instrument (Illumina) was used to detect alterations (single-nucleotide variants, multi-nucleotide variants, insertions, deletions, and gene amplifications) in assessed patient samples.

Cytokine assessments were performed via U-PLEX Biomarker Group 1 (human) 71-Plex (Meso Scale Diagnostics, Rockville, MD, USA); cytokine analytes were bound via sandwich immunoassay to biotinylated antibodies linked to U-PLEX plates as well as antibodies conjugated to electro-chemiluminescent labels (MSD GOLD™ SULFO-TAG; Meso Scale Diagnostics). A median cut-off limit of 0.356 pg/mL (limit of detection: 0.711 pg/mL) was used to distinguish between low or undetectable versus detectable or high levels of interleukin-2 (IL-2).

### Statistical analysis

All biomarker analyses were retrospective and exploratory in nature, and the number of patients in these biomarker analyses was based solely on power analyses previously performed for the clinical objectives of CHRONOS-3 and not on exploratory biomarker objectives. All analyses were performed in R (minimal version 4.0.1) [14] on all patients to evaluate biomarker efficacy associations within the treatment arms.

To quantify the association between biomarker and PFS or biomarker and OS, Kaplan–Meier plots were provided, and Cox proportional hazard models were fitted to derive *P* values and HRs with 95% confidence intervals (CIs). PTEN presence, *EZH2* or *BCL2* mutation status, and concentration levels of cytokines (low or undetectable vs. high) were considered independent variables, adding the interaction between these variables and treatment. Penalized Cox regression (Elastic Net; PFS: fivefold cross-validation and 100 repeats, OS: tenfold cross-validation and 50 repeats) was used to identify a potential combination of biomarkers and clinical variables predictive of the respective endpoint in the C + R arm. If performance was low, a multivariate regression model was fitted in accordance with the univariate approach described above, adding the top three clinical variables associated with the respective endpoint.

### Data availability

Proposals for access to anonymized patient data will be considered by the sponsor if deemed appropriate. Please contact Bayer AG at clinical-trials-contact@bayer.com regarding access.

## Results

### Patient disposition, demographics, and characteristics

**Fig. 1:**
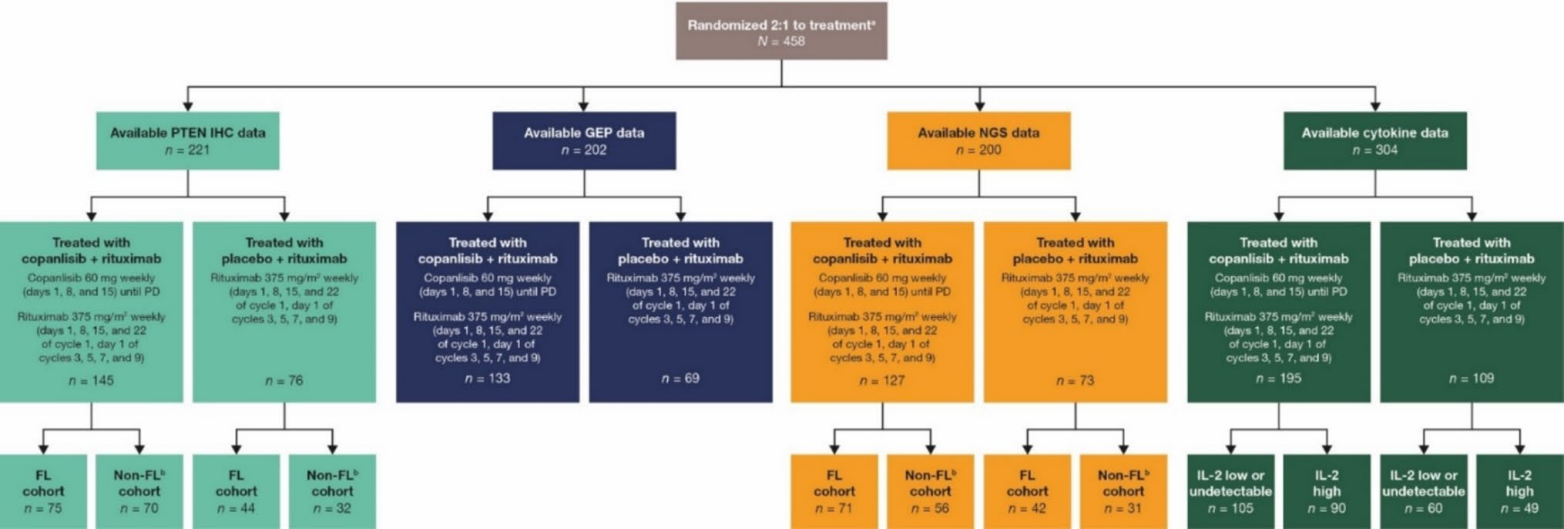
Patient disposition and biomarker eligibility. ^a^Eligible patients had a diagnosis of CD20-positive indolent B-cell lymphoma (FL grades 1–3a, marginal zone lymphoma, small lymphoplasmacytic lymphoma, or lymphoplasmacytic lymphoma/Waldenström macroglobulinemia), had relapsed following a regimen containing rituximab, a rituximab biosimilar, or an anti-CD20 monoclonal antibody, and were progression- and treatment-free for 12 months since the last rituximab-containing regimen, or for 6 months and unwilling or unfit to receive chemotherapy. ^b^The non-FL cohort included marginal zone lymphoma, small lymphoplasmacytic lymphoma, and lymphoplasmacytic lymphoma/Waldenström macroglobulinemia. *IHC* immunohistochemistry, *NGS* next-generation sequencing, *PD* progressive disease

A total of 458 patients were randomly assigned 2:1 to receive either C + R (307 patients) or P + R (151 patients) (Fig. 1). Histological subgroups in this study included FL (*n* = 275), MZL (*n* = 95), SLL (*n* = 50), and LPL/WM (*n* = 38), and all enrolled patients had an Eastern Cooperative Oncology Group performance status of 2 or below. The median age was 63 years (range 54–70) in the C + R arm and 62 years (range 53–70) in the P + R arm. The percentage of patients in the C + R arm and the P + R arm who had already received two or more anticancer therapies was 51.1% (*n* = 157) and 53.0% (*n* = 80), respectively.

### Biomarker analyses

#### PTEN immunohistochemistry analyses

To investigate the relationship between PTEN presence and patient response to treatment with respect to PTEN expression was assessed via immunohistochemistry staining in patients where tissue was available (*N* = 221/307). PTEN presence was observed in 81 out of 221 (36.7%) patients with iNHL, with a similar PTEN percent positivity in the FL cohort (*n* = 41/119, 34.5%; Supplementary Table S2).

**Fig. 2:**
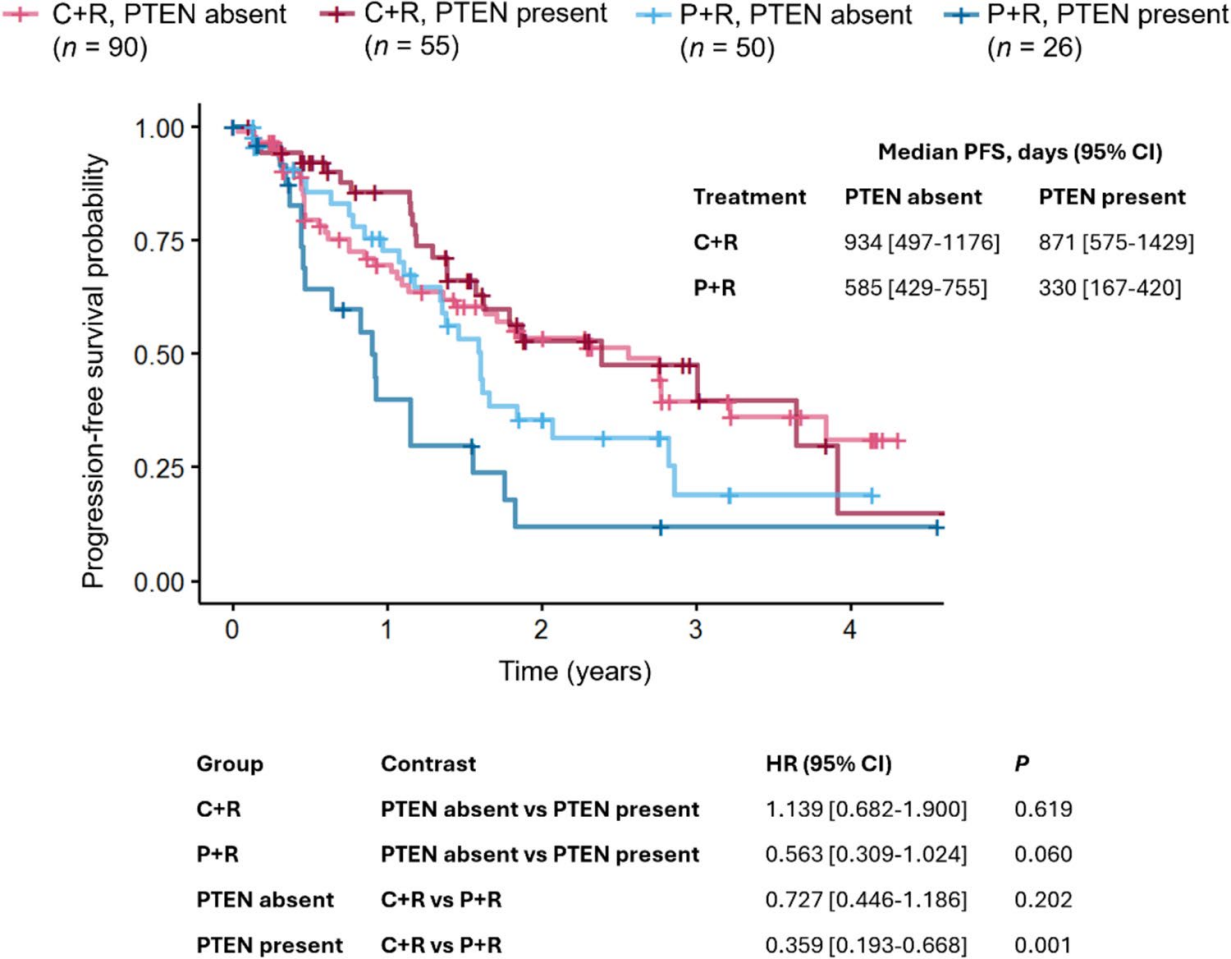
PFS by PTEN status in patients with iNHL. *C* copanlisib, *P* placebo, *R* rituximab

**Fig. 3:**
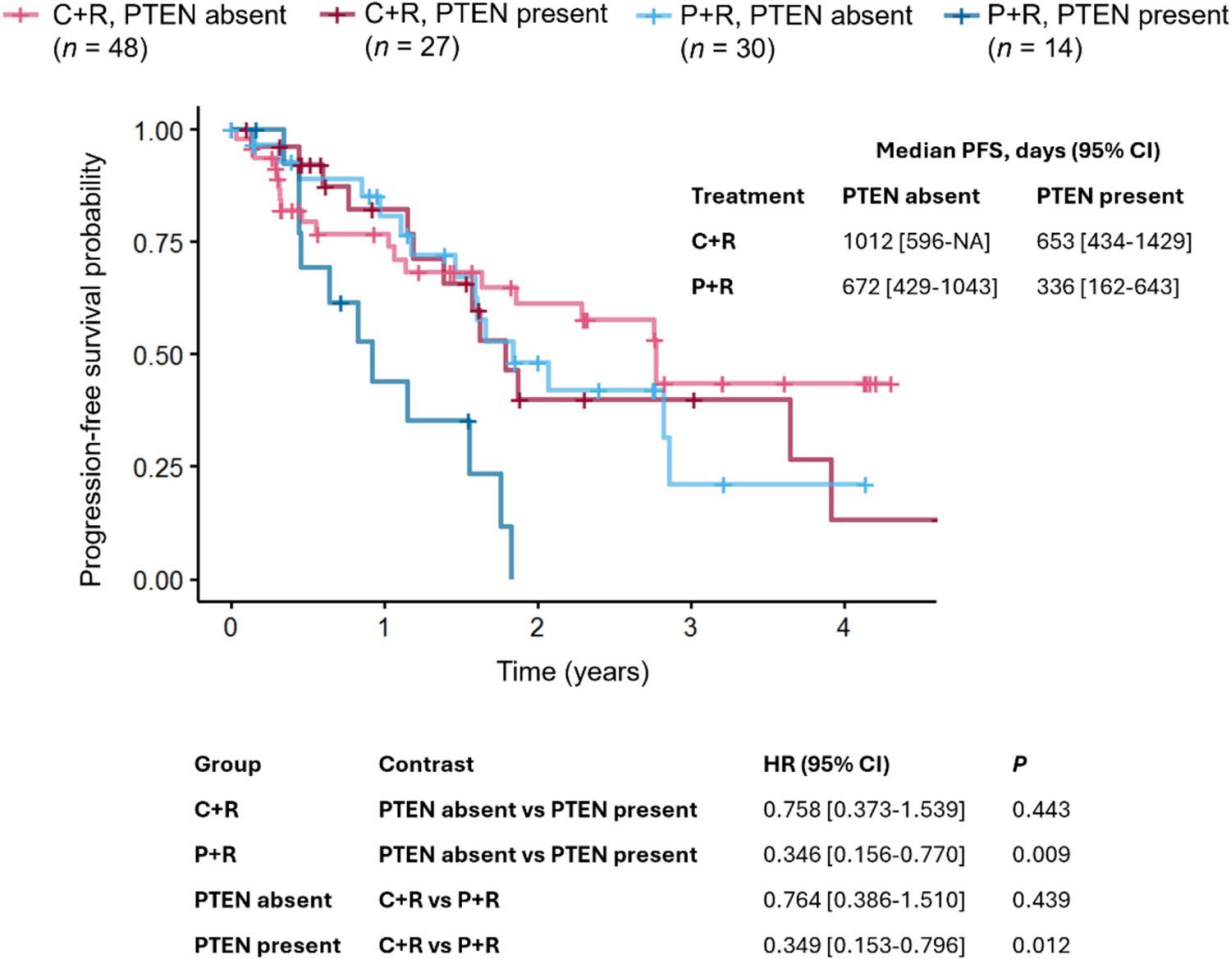
PFS by PTEN status in patients with FL. *C* copanlisib, *NA* not available, *P* placebo, *R* rituximab

In patients with iNHL and the sub-cohort of FL, an improved median PFS benefit was observed for the C + R arm compared with the P + R arm, regardless of PTEN presence (Figs. 2, 3). In the P + R arm, PTEN absence was associated with significant improvements in PFS compared with PTEN presence in the FL cohort (*P* = 0.009; HR 0.346 [95% CI 0.156–0.770]), and a similar trend was seen in patients with iNHL (*P* = 0.060; HR 0.563 [95% CI 0.309–1.024]), but not in the non-FL arm (Supplementary Fig S1). As a result of this enhanced PFS benefit in patients with PTEN absence, PFS was not significantly improved for C + R over P + R in patients with PTEN absence (*P* = 0.202; HR 0.727 [95% CI 0.446–1.186] in patients with iNHL; *P* = 0.439; HR 0.764 [95% CI 0.386–1.510] in the FL cohort); however, significant improvements were observed for C + R compared with P + R in patients with PTEN presence (*P* = 0.001; HR 0.359 [95% CI 0.193–0.668] in patients with iNHL; *P* = 0.012; HR 0.349 [95% CI 0.153–0.796] in the FL cohort). No multivariate analysis was conducted because none of the clinical variables were significantly associated with PFS in the subset of patients with available PTEN measurements upon consideration of the adjusted *P* value. The relationship between OS and PTEN presence was also investigated, but due to a limited number of events, no statistical difference was observed between patients with PTEN presence versus PTEN absence (Supplementary Fig. S2).

#### EZH2 and BCL2 next-generation sequencing analyses

**Fig. 4:**
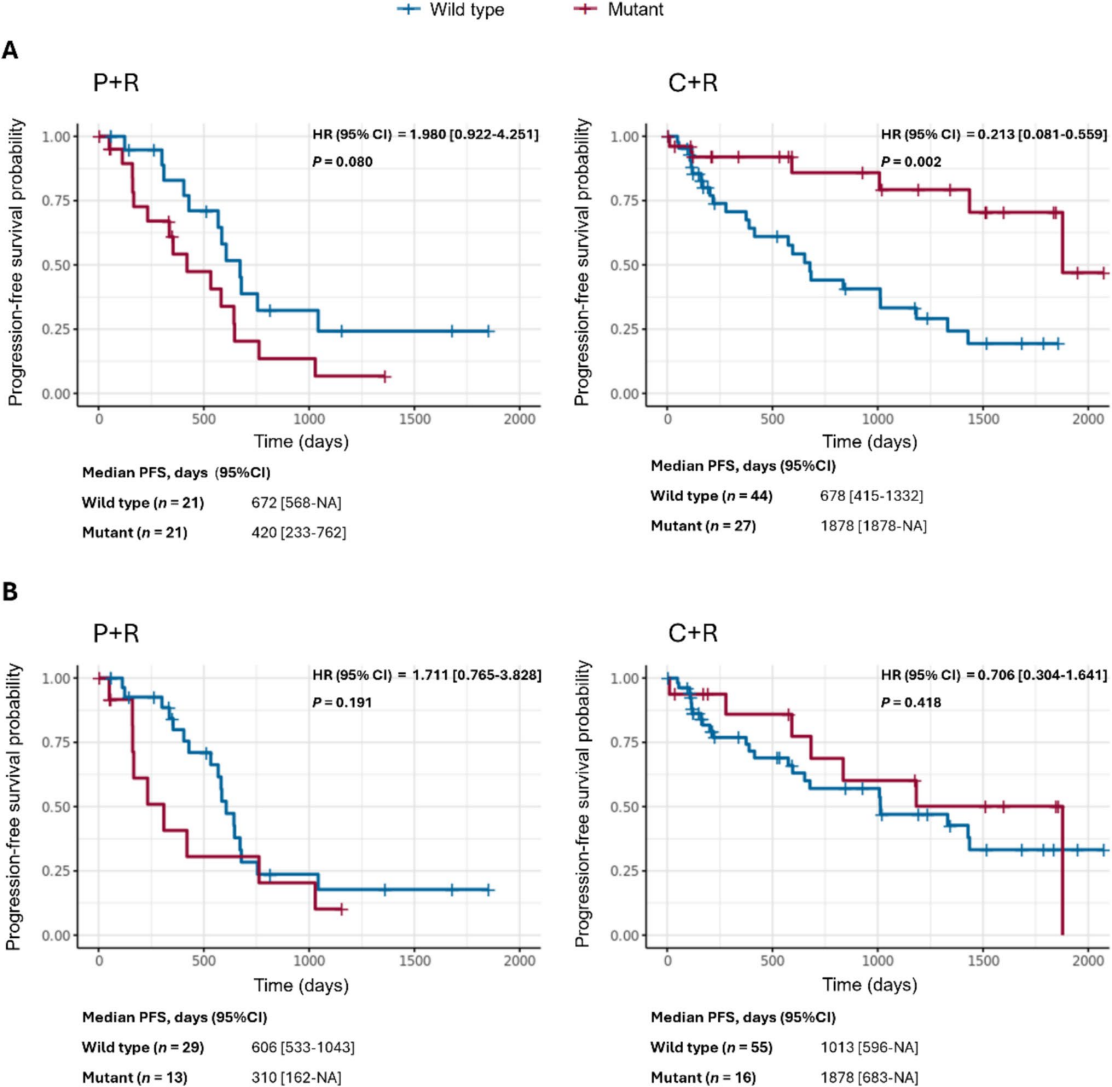
Association of **A** *BCL2* mutation status and **B** *EZH2* mutation status with PFS response by treatment in patients with FL. *C* copanlisib, *NA* not available, *P* placebo, *R* rituximab

Targeted DNA sequencing using the TruSight Oncology 500 Assay was performed to assess the mutation status of genes within the 523 gene panel, which were subsequently tested retrospectively for association with patient response to treatment. This data was available for a total of 200 iNHL patients at baseline/screening, of which 113 were FL lymphoma patients. The genes mutated in over 25% of the iNHL population were also mutated in over 25% of the FL subgroup, and these include *BARD1, CREBBP, SLX4* and *BCL2*. Within the FL population, an additional high prevalence of mutations is observed within *TNFRSF14, ICOSLG* and *EZH2*. Of these top mutated genes, *BCL2* was the only gene that showed a significant difference between mutant and wild type when treated with copanlisib and rituximab. The identification of mutant *BCL2* in this panel reflects previous investigations into the genomic landscape of FL, where *BCL2* is known to be commonly mutated in FL [15, 16]. Mutations in *BCL2* were detected in 51 out of 200 (25.5%) iNHL patients, and the majority of these *BCL2* mutations (48 out of 51) patients were in the FL sub-group. With C + R treatment, PFS was significantly improved in FL patients with *BCL2* mutations relative to those with wild-type *BCL2* (*P* = 0.002; HR 0.213 [95% CI 0.081–0.559]) (Fig. 4A). In FL patients treated with P + R, no significant difference in PFS based on *BCL2* mutation status was observed (*P* = 0.080; HR 1.980 [95% CI 0.922–4.251]). Detected *BCL2* mutations in FL patients were distributed throughout the *BCL2* gene, and no hotspot mutations were identified. The most prevalent *BCL2* mutations were G47D (*n* = 5) and G47A (*n* = 4) missense mutations (Supplementary Fig. S3) with a frequency of 8.7% (*n* = 9/103).

There has been a growing area of interest in cancer treatment to target epigenetic regulators that modify gene expression, dysregulation of which can lead to tumorigenesis. One such target that has been studied is enhancer zeste homolog 2 (*EZH2*), which can develop mutations affecting its activity. These mutations are mostly seen in germinal center (GC) derived diffuse large B-cell lymphoma and FL because *EZH2* plays a major role in GC development and is greatly expressed within pro-B cells, which directly controls *IGF1R* expression to activate the PI3K pathway, therefore making it an interesting gene to evaluate in this study. Mutations in *EZH2* are typically found in approximately 20% of FL patients, and in this CHRONOS-3 study they were detected in 29 out of 113 FL (25%) patients.

In the FL cohort, when treated with P + R, patients with mutant *EZH2* appear to have a worse PFS as compared to the wild-type *EZH2* patients. However, patients treated with C + R not only show much improved median PFS as compared to the placebo group but also a comparable PFS with both the wild-type and the mutant forms of *EZH2* (*P* = 0.418; HR 0.706 [95% CI 0.304–1.641]) (Fig. 4B) indicating that inhibition of the PI3K pathway by copanlisib provides the same PFS advantage to both the mutant and wt *EZH2*.

Cross-validated penalized Cox regression was used to identify a potential combination of genes and clinical variables predictive of PFS in the C + R arm. In FL patients, *BCL2* was the only gene associated with PFS, with mutant *BCL2* providing a benefit over wt *BCL2*, while no difference in PFS was observed between mutant and wt *EZH2* (Supplementary Table S3). In addition, tumor stage and geographic region were associated with PFS (Supplementary Table S3; predictive performance: mean C-index 0.90 [95% CI 0.80–1.00]).

#### IL-2 cytokine analysis

**Fig. 5:**
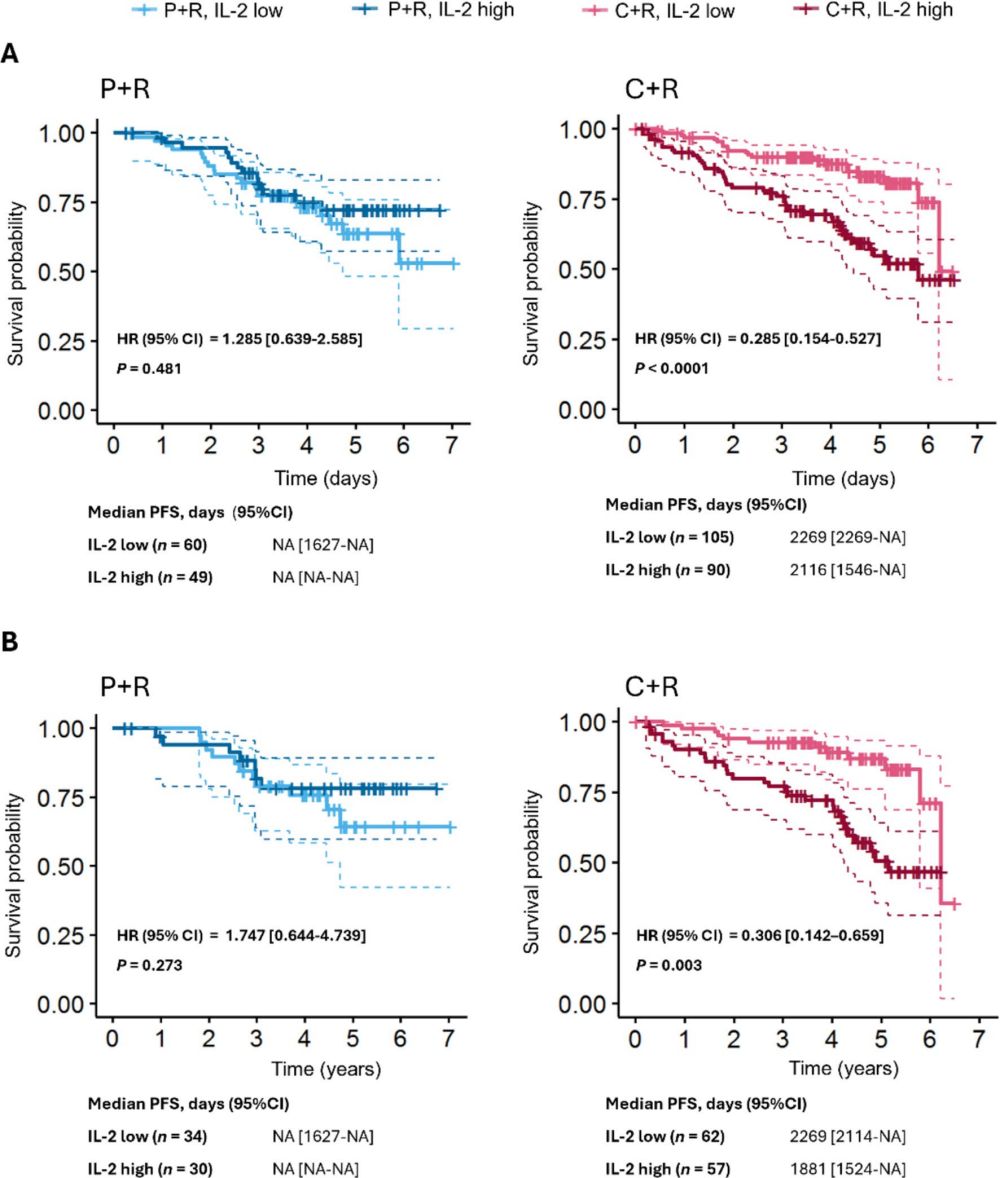
OS by baseline levels of IL-2 in **A** patients with iNHL and **B** patients with FL. ***C*** copanlisib, *NA* not available, *P* placebo, *R* rituximab

As the pan-PI3K activity of copanlisib may induce responses across various immune cell subtypes during development and activation, a full array of 71 cytokines and chemokines was used to examine the association between biomarker levels and patient response. As a follow-up suggestion from the ODAC, it is important to not only look at PFS, the primary endpoint of the trial but also at the OS, the secondary endpoint. Though none of the analyzed cytokines and chemokines at baseline showed any association with PFS upon consideration of the adjusted *P* value (Supplementary Table S4), the association with OS was significant with baseline levels of IL-2. Here, we present results detailing the relationship between baseline IL-2 levels and OS in 304 evaluable patients, as this was the cytokine associated with the largest OS benefit (Supplementary Table S4). In the C + R arm, a significant OS benefit (unadjusted *P* value) was observed for patients with low or undetectable (≤ 0.356 pg/mL) baseline levels of IL-2 versus those with high IL-2 levels in patients with iNHL (*P* < 0.0001; HR 0.285 [95% CI 0.154–0.527]) (Fig. 5A) and the subset of the FL cohort (*P* = 0.003; HR 0.306 [95% CI 0.142–0.659]) (Fig. 5B) and the non-FL cohort (Supplementary Fig. S4). No significant difference in OS was demonstrated between patients with low and high IL-2 expression when treated with P + R in either patients with iNHL (*P* = 0.481; HR 1.285 [95% CI 0.639–2.585]) (Fig. 5A) or the FL cohort (*P* = 0.273; HR 1.747 [95% CI 0.644–4.739]) (Fig. 5B).

A combination of cytokines and clinical variables selected via cross-validated penalized Cox regression based on OS had low predictive performance in iNHL patients (Mean C-index 0.77 [95% CI 0.51–0.94]) so no meaningful conclusions could be drawn. A multivariate Cox regression model was fitted which included the top three clinical variables (ECOG grade, bulky disease and tumor size) associated with OS as covariates. After adjusting for the clinical variables, a significant OS benefit was observed for patients with low or undetectable baseline levels of IL-2 versus those with high IL-2 levels, whereas no significant difference in OS was demonstrated between patients with low and high IL-2 expression when treated with P + R (Supplementary Table S5). Patients without bulky disease and with ECOG grade 0 showed longer OS than patients with bulky disease and ECOG grade ≥ 1, while tumor size did not affect OS (Supplementary Table S5).

## Discussion

Here, we have described the exploratory analysis of biomarker data in patients with relapsed B-cell iNHL, which seeks to identify biomarkers that may correlate to patient response following treatment with C + R.

PTEN, a tumor suppressor protein that negatively regulates the PI3K pathway, is known to be one of the most commonly mutated genes in human cancer [17]. Complete loss of PTEN protein expression leads to aberrant PI3K activation and signaling, associated with poor patient outcomes [18]. This study is one of the largest in hematological malignancies to evaluate the relationship between PTEN presence and patient response to C + R treatment. Early biomarker reports of copanlisib suggested that response could be enhanced in patients with iNHL and PTEN absence [19], but here baseline PTEN presence had no impact on PFS with C + R, which may be due to the high response rates seen in this study. This finding, that there is a higher PFS in PTEN-negative patients may seem counterintuitive because of the role of PTEN as a tumor suppressor gene. One explanation may relate to PTEN maintenance of B-cell homeostasis. In iNHL malignancies, cells may be addicted to the constitutively active Bruton’s tyrosine kinase (BTK) pathway and thus require a fine balance between PI3K and BTK signaling for optimal B-cell survival. Absence of functional PTEN expression may increase levels of phosphatidylinositol (3,4,5)-trisphosphate, leading to hyperactivation of the BTK and PI3K pathways. As the PI3K pathway is involved in cell metabolism and nutrient regulation [20], these cells may then be unable to meet their subsequent nutrient demands, challenging B-cell survival and subsequently improving PFS. PTEN expression might also influence the tumor microenvironment in ways that promote immune escape, or in the presence of PTEN, suppression of PI3K signaling may result in the activation of compensatory, linked signaling pathways that could bypass the inhibition of PI3K and promote cell survival [21]. Since the PTEN gene is susceptible to both deletions and mutations, it was essential to explore the implications of PTEN gene alterations which could guide future research and influence clinical response. However, there were only 2/200 samples with PTEN mutations observed in this data set, making a comprehensive analysis difficult. It is important to note the speculative nature of these explanations, and the exact mechanisms are likely to be complex and multifactorial. Further research is needed to elucidate the precise biological reasons behind this observation and determine treatment strategies for iNHL patients.

Translocation t(14;18) of *BCL2*, a gene that encodes a negative regulator of apoptosis, is a key driver of FL pathogenesis in over 90% of FL cases [22]. *BCL2* is further susceptible to mutations in the coding sequence of the gene, which are common in FL and may be associated with impaired apoptosis, as well as an increased risk of transformation to an aggressive lymphoma and death [12]. In a recent study, *BCL2* mutations were observed in 72.5% of FL patients (allele frequency of > 1%) [15], which is higher than the reported prevalence of *BCL2* mutations in the CHRONOS-3 study. Of the total *BCL2* mutations (*N* = 51) in the study, 94% were in the FL subset and mutations were spread across the length of the *BCL2* gene, as observed in similar analyses [12]. While studies have reported conflicting data on whether *BCL2* mutation status correlates with survival in FL patients [15, 16, 23], the presence of *BCL2* mutations in this analysis was associated with improved PFS in patients with FL treated with C + R compared with P + R.

EZH2, a histone methyltransferase, promotes transcriptional gene silencing by catalyzing histone methylation [24, 25]. Sequencing studies have demonstrated that *EZH2* mutations are common in FL [13] and are believed to be a potential driver of lymphomagenesis [26]. Furthermore, gain-of-function mutations in *EZH2* promote B-cell proliferation and survival [24], warranting their evaluation within this study. Here, *EZH2* mutations were reported in 41% of FL patients, which is slightly higher than the literature-reported prevalence (> 25%) [13]. More data are needed to inform how *EZH2* mutation status relates to treatment outcomes [27]; however, recent data suggest that *EZH2* mutations in FL patients may be predictive of improved response to rituximab and chemotherapy [27]. The exact reason why *EZH2* mutations did not impact the PFS significantly is unknown, but the suggested reasoning is that activated AKT phosphorylates EZH2 leading to tumor growth. Since copanlisib inhibits the AKT pathway, which is upstream of EZH2 phosphorylation, treatment with C + R seems to benefit both arms equally.

IL-2 is a key cytokine involved in immune system homeostasis and T-cell maintenance and was previously approved by the FDA as immunotherapy for metastatic renal cell carcinoma and metastatic melanoma [28]. However, the clinical utility of IL-2 as an anti-tumor therapeutic is limited by reports of severe toxicity and an opposing role of IL-2 in both effector and regulatory T-cell signaling [28]. Here, a significant OS benefit for patients with low or undetectable baseline levels of IL-2 over patients with high baseline levels of IL-2 was observed in patients with iNHL treated with C + R. IL-2 differentially drives the fate of CD4 + and CD8 + T cells; high levels of IL-2 signaling promote differentiation into short-lived effector cells, while low levels of IL-2 signaling promote memory T-cell differentiation [29]. Therefore, low levels of IL-2 at baseline may result in the preferential expansion of long-lived memory CD4 + T cells but not CD8 + cytotoxic T-cell populations, thus potentially having an impact on anti-tumor activity and OS. The discovery that a subset of patients with low baseline levels of IL-2 demonstrate enhanced OS benefit from copanlisib is significant, as it may suggest that this subgroup may see particularly beneficial improvements in survival from PI3K treatment. This finding is timely and relevant, given an ODAC meeting on April 21, 2022, to discuss PI3K class-wide findings, as well as published guidance from the FDA that requests the collection and analysis of OS data for PI3K trials [30].

Understanding the cumulative impact of PTEN expression, EZH2 and BCL2 mutations, and cytokine levels can provide valuable prognostic insights. The interplay among these biomarkers can be complex and a patient may exhibit a unique treatment response profile informing clinical decision-making and enabling personalized approaches that account for the multifaceted nature of these biomarkers and their collective influence on patient responses to therapy, providing valuable prognostic insights. Patients exhibiting a specific combination of these biomarkers may be stratified into different risk categories, guiding treatment decisions and monitoring strategies. A complete multivariate analysis was limited due to the lack of availability of a complete data set. The data presented here demonstrate the association of several biomarker signals with improved patient response and survival following treatment with C + R, suggesting potential predictive utility in treatment selection, but these results are limited by the exploratory nature of these analyses. While the reported associations require further investigation, these results serve as a foundation for future research into predictive biomarkers for the effective management of iNHL.

## Supplementary Information

Below is the link to the electronic supplementary material.

Supplementary file1 (DOCX 585 kb)
