# Supplementary material for: Exploratory biomarker analysis from a phase III study of the PI3K inhibitor, copanlisib, in combination with rituximab in patients with indolent non-Hodgkin lymphoma, a retrospective study
Source: Clin Transl Oncol. 2025 Feb 21;27(8):3439–48. doi: 10.1007/s12094-025-03869-2 (PMC12259725; doi:10.1007/s12094-025-03869-2)
Supplement: Supplementary file 1 — Supplementary file1 (DOCX 585 kb) [file 12094_2025_3869_MOESM1_ESM.docx]

**Supplementary Figure S1.** PFS by PTEN status in patients with non-FL.


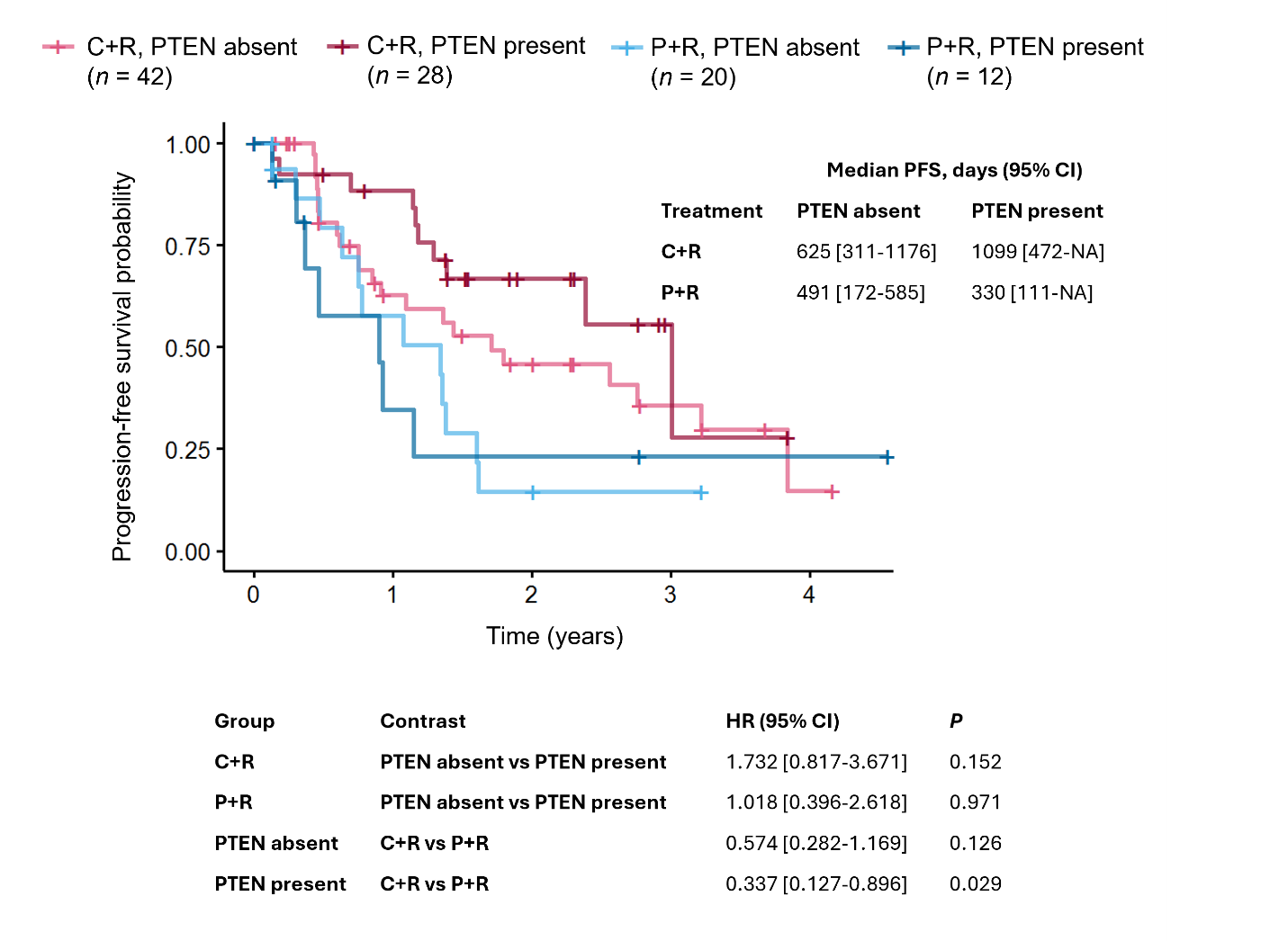

C, copanlisib; NA, not available; P, placebo.

**Supplementary Figure S2.** OS by PTEN status in patients with iNHL.


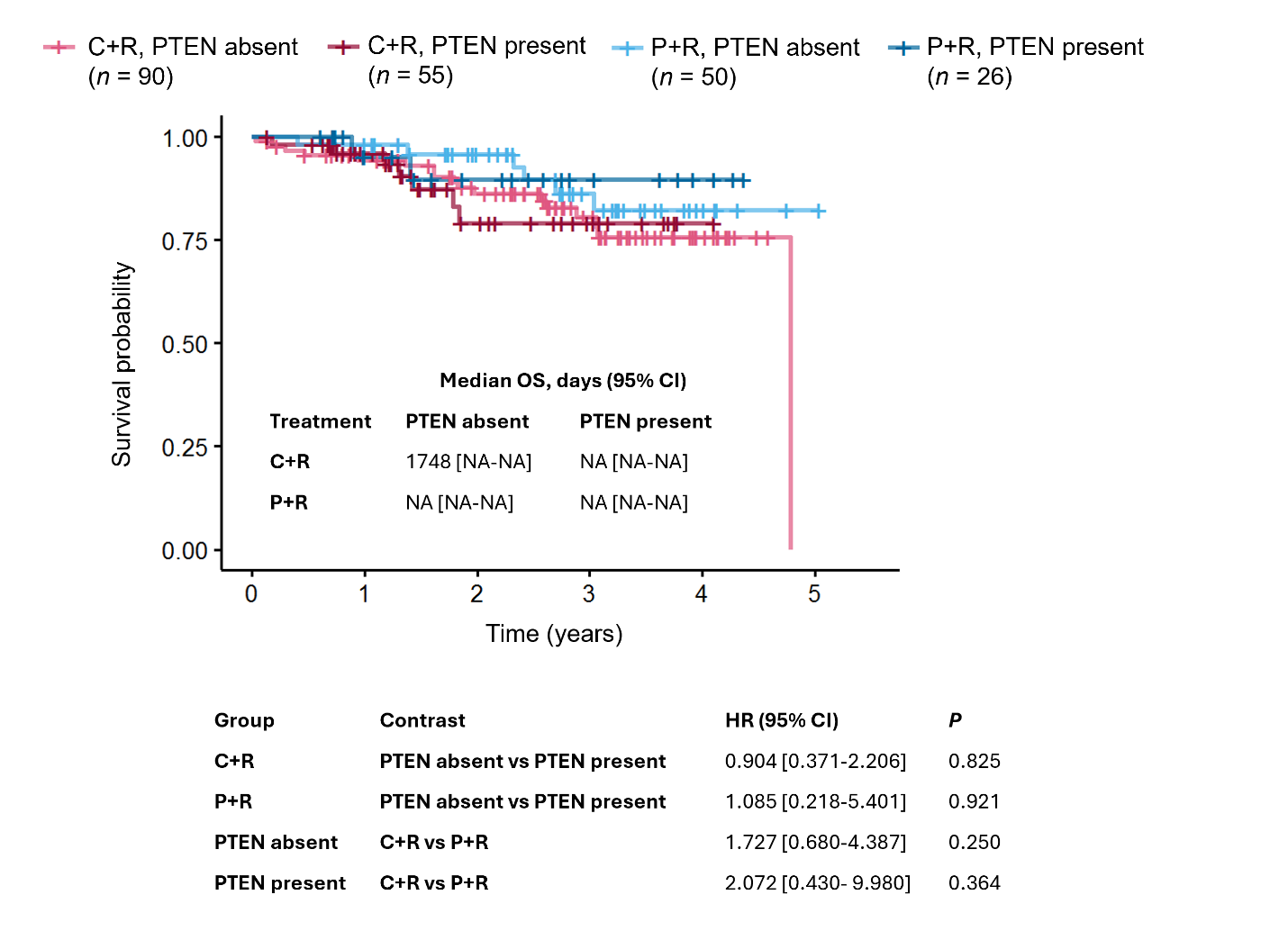


C, copanlisib; NA, not available; P, placebo; R, rituximab.

**Supplementary Figure S3.** Lollipop plot of *BCL2* mutations in patients with FL.


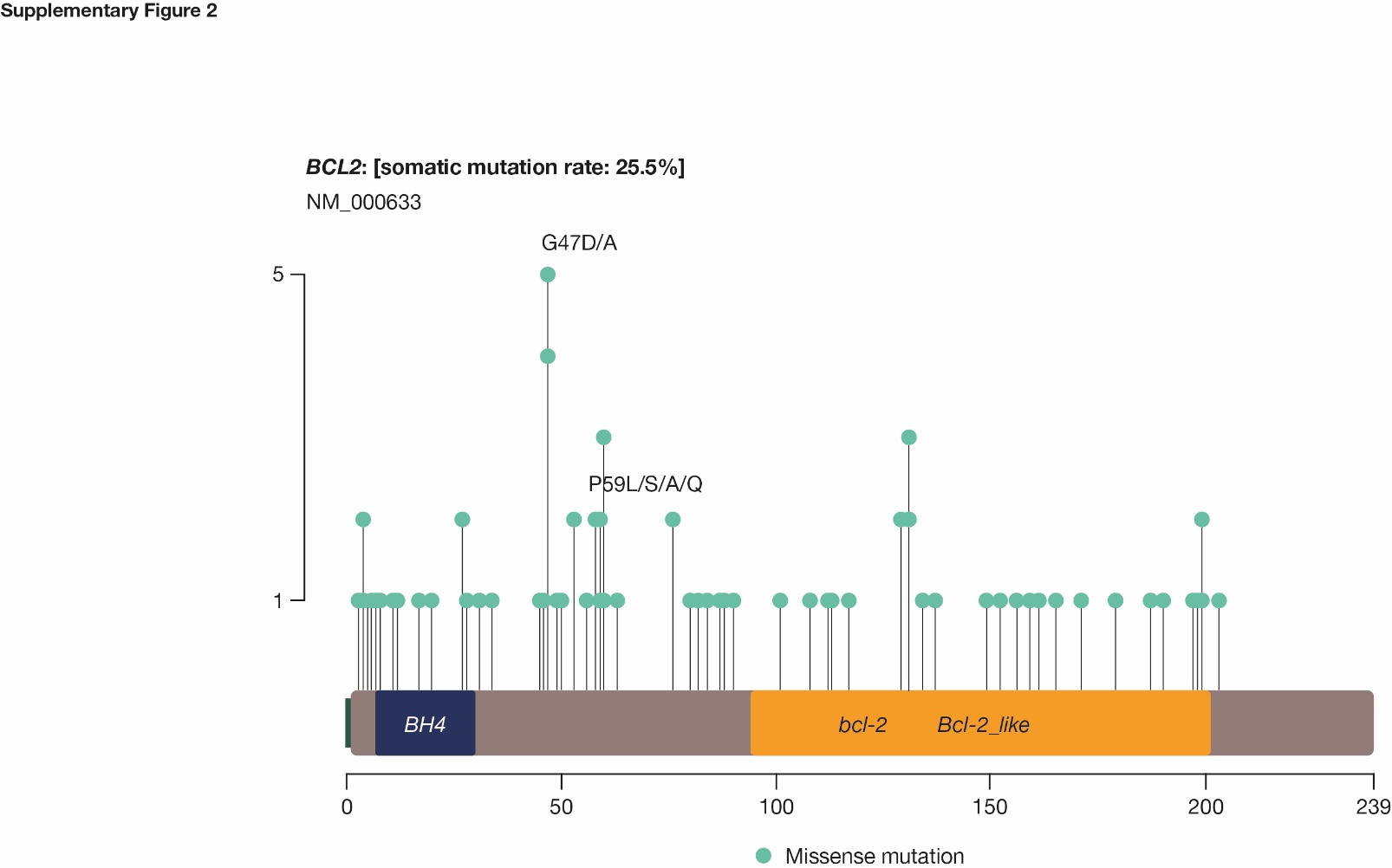


**Supplementary Figure S4.** OS by baseline levels of IL-2 in patients with non-FL.


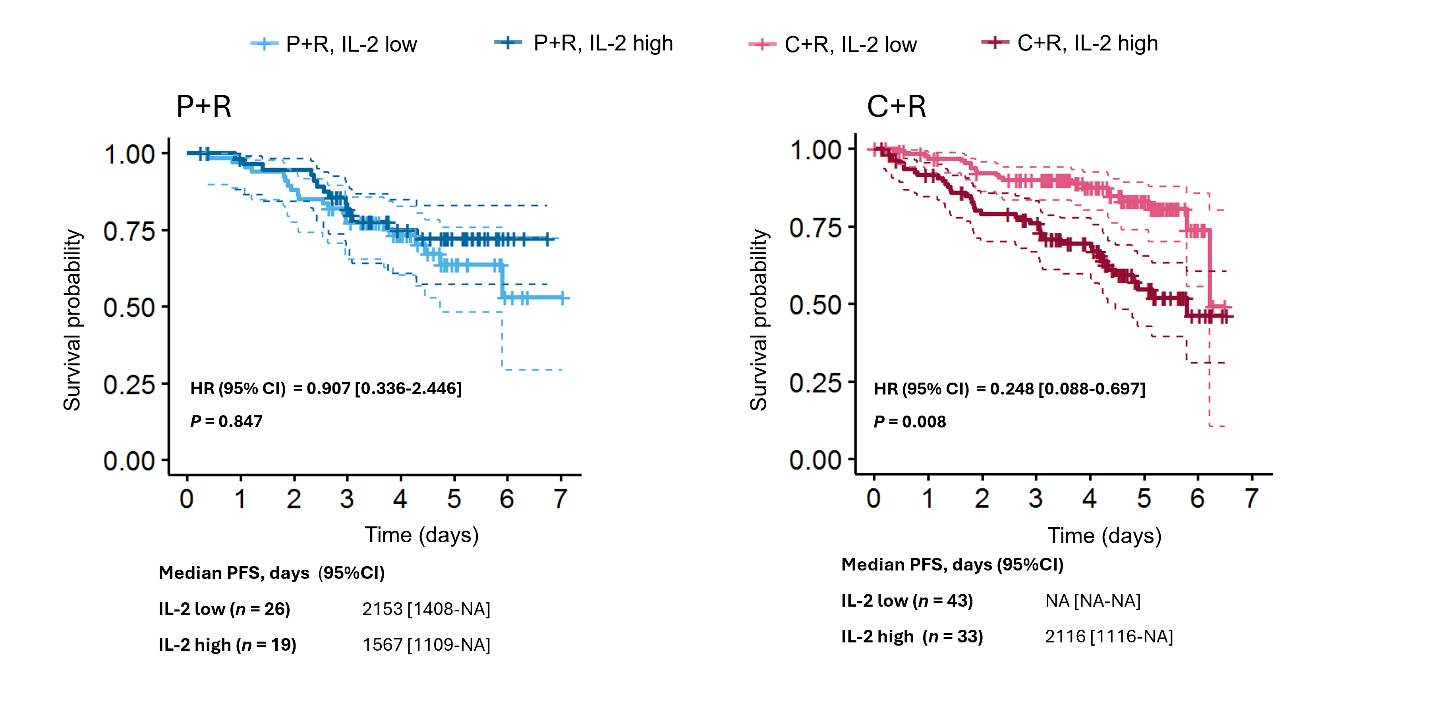


C, copanlisib; NA, not available; P, placebo; R, rituximab.

**Supplementary Table S1.** Representativeness of CHRONOS-3 participants.

| **Cancer type, subtype, stage, condition** | Relapsed or refractory iNHL (follicular lymphoma grades 1–3a, lymphoplasmacytic lymphoma/Waldenström macroglobulinemia, marginal zone lymphoma, small lymphocytic lymphoma) with at least two lines of therapy (1) |
| --- | --- |
| **Considerations related to:** | |
| **Sex** | Incidence rates for NHL are higher for men than women (2), and the cumulative lifetime risk of developing NHL in men (0.72%) is more than twice the risk in women (0.35%) (3). Similarly, the reported cumulative lifetime risk of mortality due to NHL is 0.33% in men compared with 0.21% in women (3) |
| **Age** | A majority of patients (57%) diagnosed with NHL are aged >65 years, and the average age at diagnosis is 67 years (3). However, certain subtypes of NHL are more likely to present in younger patients, including Burkitt’s lymphoma, diffuse large B-cell lymphoma, lymphoplasmacytic lymphoma, anaplastic large cell lymphoma, and marginal zone lymphoma (3) |
| **Race/ethnicity** | In the USA, the greatest risk of NHL by race or ethnicity is in White and non-Hispanic populations (3). Five-year incidence rates (adjusted by age) are 20.1 and 17.2 per 100,000 in White and Hispanic populations, respectively, compared with 15.0, 14.3, and 13.4 per 100,000 in American Indian/Alaska Native, Black, and Asian/Pacific Islander populations, respectively (4) |
| **Geography** | Globally, age-adjusted incidence rates for NHL are highest in Australia and New Zealand (12.5 per 100,000), North America (12.0 per 100,000), Northern Europe (11.4 per 100,000), and Western Europe (10.2 per 100,000) (2). Mortality from NHL is highest in parts of Africa, Western Asia, and Oceania (2) |
| **Other considerations** | In the USA, patients from Black and Hispanic racial backgrounds and female patients are significantly under-represented in randomized controlled trials in lymphoma, while White and male patients are over-represented (5) |
| **Overall representativeness of this study** | The age distribution of patients in CHRONOS-3 (median age, 63 years [range, 28–91]) (1) is similar to the average age distribution of iNHL in the literature (3) |
|  | CHRONOS-3 was conducted in 186 academic medical centers across the world, including Asia, Australia, Europe, New Zealand, North America, Russia, South Africa, and South America (1). 38.2% of patients enrolled in CHRONOS-3 were from the Asia-Pacific region, which is in line with the proportion of new annual NHL cases from Asia (44.4%) and Oceania (1.4%) (1,2). Similarly, patients from Europe represented 32.1% of the CHRONOS-3 population and 22.6% of new global cases each year (1,2), suggesting that CHRONOS-3 was fairly representative of the global NHL population |
|  | The CHRONOS-3 patient population was fairly balanced between men (52.0%) and women (48.0%) (1), which does not reflect the higher global incidence rate of NHL in men compared with women (2) |

**Supplementary Table S2.** PTEN expression observed in patients with iNHL and the FL cohort.

| **PTEN expression** | **iNHL (*N* = 221)** | **FL (*n* = 119)** |
| --- | --- | --- |
| Presence, *n* (%) [copanlisib/placebo] | 81 (36.7) [55/26] | 41 (34.5) [27/14] |
| Absence, *n* (%) [copanlisib/placebo] | 140 (63.3) [90/50] | 78 (65.6) [48/30] |

**Supplementary Table S3.** Results from cross-validated penalized Cox regression analysis of the association between PFS and mutation status and clinical variables in the C+R arm.

| Variable | Level vs. reference level | Mean HR  [95% CI]^a^ |
| --- | --- | --- |
| Tumor stage | - | 1.377 [1.127-1.682] |
| *BCL2* | Mutant vs. WT | 0.379 [0.236-0.608] |
| Geographic region | Africa/Australasia/Russia/South America/Turkey vs. Asia Pacific | 1.442 [1.001-2.077] |
| Diabetes | Yes vs. No | 1.706 [0.939-3.102] |
| *TP53* | Mutant vs. WT | 1.306 [0.891-1.914] |
| *EP300* | Mutant vs. WT | 1.207 [0.873-1.670] |
| Renal function | Normal vs. Mild impairment | 0.857 [0.649-1.131] |
| Ethnicity | Not Hispanic or Latino vs. Hispanic or Latino | 0.857 [0.599-1.226] |
| *CHEK2* | Mutant vs. WT | 0.855 [0.616-1.185] |
| FLIPI | Low vs. High | 0.856 [0.598-1.224] |
| Race | Asian vs. American Indian or Alaska Native | 0.914 [0.742-1.126] |
| AST | - | 0.991 [0.969-1.013] |
| *ICOSLG* | Mutant vs. WT | 0.944 [0.791-1.127] |
| *LRP1B* | Mutant vs. WT | 0.941 [0.773-1.146] |
| *EZH2* | Mutant vs. WT | 0.959 [0.793-1.160] |
| Renal function | Moderate impairment vs. Mild impairment | 1.058 [0.846-1.323] |
| Systemic therapy | 1 line vs. ≥4 lines | 0.965 [0.822-1.132] |
| *ARID1B* | Mutant vs. WT | 0.972 [0.843-1.121] |
| Age | - | 1.001 [0.997-1.005] |
| Race | White vs. American Indian or Alaska Native | 1.017 [0.924-1.120] |
| Systemic therapy | 2 lines vs. ≥4 lines | 1.019 [0.900-1.154] |
| Hypertension | Yes vs. No | 1.010 [0.943-1.082] |
| Sex | Male vs. Female | 1.017 [0.899-1.151] |
| *FAT1* | Mutant vs. WT | 1.016 [0.906-1.140] |
| Tumor size | - | 1.000 [0.999-1.001] |
| Smoking history | Former vs. Current | 1.023 [0.854-1.226] |
| Lesion number | - | 1.000 [0.997-1.003] |
| *TNFRSF14* | Mutant vs. WT | 1.009 [0.923-1.104] |
| Bulky disease | Yes vs. No | 1.010 [0.919-1.111] |
| *SOCS1* | Mutant vs. WT | 1.011 [0.925-1.105] |
| *NOTCH1* | Mutant vs. WT | 1.003 [0.972-1.035] |
| Bilirubin | - | 0.993 [0.903-1.091] |
| Hepatic function | Normal vs. Mild impairment | 1.013 [0.875-1.174] |
| *BARD1* | Mutant vs. WT | 0.996 [0.936-1.060] |
| *FAS* | Mutant vs. WT | 0.997 [0.957-1.039] |
| BMI | - | 1.000 [0.997-1.003] |
| Previous PI3K therapy | Yes vs. No | 1.002 [0.977-1.027] |
| *SLX4* | Mutant vs. WT | 1.000 [0.995-1.004] |
| *KAT6A* | Mutant vs. WT | 0.998 [0.972-1.024] |
| *TET1* | Mutant vs. WT | 1.003 [0.945-1.065] |
| *NOTCH3* | Mutant vs. WT | 1.000 [0.994-1.007] |
| Entry criterion | Unfit for chemotherapy vs. treatment-free interval | 1.000 [0.999-1.001] |
| FLIPI | Intermediate vs. High | 1.005 [0.918-1.101] |
| Geographic region | Europe vs. Asia Pacific | 1.000 [0.996-1.004] |
| Systemic therapy | 3 lines vs. ≥4 lines | 1.000 [0.996-1.004] |
| *ZFHX3* | Mutant vs. WT | 1.000 [0.991-1.008] |
| ECOG |  | 1.000 [1.000-1.000] |
| Geographic region | North America vs. Asia Pacific | 1.000 [1.000-1.000] |
| Hepatic function | Moderate impairment vs. Mild impairment | 1.000 [1.000-1.000] |
| Race | Black or African American vs. American Indian or Alaska Native | 1.000 [1.000-1.000] |
| Smoking history | Never vs. Current | 1.000 [1.000-1.000] |
| Viral infection | Yes vs. No | 1.000 [1.000-1.000] |
| *CREBBP* | Mutant vs. WT | 1.000 [1.000-1.000] |
| *ARID1A* | Mutant vs. WT | 1.000 [1.000-1.000] |
| *CARD11* | Mutant vs. WT | 1.000 [1.000-1.000] |

^a^Mean HR and 95% CI across cross-validation folds and repeats

**Supplementary Table S4.** Predictive value of biomarkers for PFS and OS.

|  |  | **Cox regression (PFS)^a^** | | **Cox regression (OS)^b^** | |
| --- | --- | --- | --- | --- | --- |
| **Biomarker** | **Function** | **Raw interaction  *P* value** | **Adjusted interaction  *P* value** | **Raw interaction  *P* value** | **Adjusted interaction  *P* value** |
| CTACK | Chemokines | 0.008 | 0.220 | 0.041 | 0.797 |
| ENA-78 | Chemokines | 0.738 | 0.878 | 0.501 | 0.936 |
| Eotaxin | Chemokines | 0.182 | 0.524 | 0.875 | 0.971 |
| Eotaxin-2 | Chemokines | 0.331 | 0.618 | 0.355 | 0.797 |
| Eotaxin-3 | Chemokines | 0.247 | 0.544 | 0.944 | 0.999 |
| EPO | Other cytokines | 0.389 | 0.690 | 0.913 | 0.997 |
| FLT3L | Other cytokines | 0.133 | 0.524 | 0.247 | 0.797 |
| Fractalkine | Chemokines | 0.147 | 0.524 | 0.501 | 0.936 |
| G-CSF | Other cytokines | 0.565 | 0.756 | 0.679 | 0.971 |
| GM-CSF | Other cytokines | 0.686 | 0.870 | 0.284 | 0.797 |
| GROα | Chemokines | 0.601 | 0.790 | 0.874 | 0.971 |
| I-309 | Chemokines | 0.009 | 0.220 | 0.662 | 0.971 |
| IFNα2a | Other cytokines | 0.421 | 0.712 | 0.866 | 0.971 |
| IFNβ | Other cytokines | 0.979 | 0.979 | 0.339 | 0.797 |
| IFNγ | Other cytokines | 0.005 | 0.220 | 0.305 | 0.797 |
| IL-10 | Anti-inflammatory | 0.550 | 0.751 | 0.294 | 0.797 |
| IL-12/IL-23p40 | Pro-inflammatory | 0.132 | 0.524 | 0.983 | 0.999 |
| IL-12p70 | Pro-inflammatory | 0.834 | 0.913 | 0.755 | 0.971 |
| IL-13 | Anti-inflammatory | 0.173 | 0.524 | 0.242 | 0.797 |
| IL-15 | Other cytokines | 0.869 | 0.918 | 0.740 | 0.971 |
| IL-16 | Other cytokines | 0.836 | 0.913 | 0.370 | 0.797 |
| IL-17A | Pro-inflammatory | 0.168 | 0.524 | 0.236 | 0.797 |
| IL-17A/F | Pro-inflammatory | 0.298 | 0.588 | 0.490 | 0.936 |
| IL-17B | Pro-inflammatory | 0.814 | 0.913 | 0.345 | 0.797 |
| IL-17C | Pro-inflammatory | 0.526 | 0.751 | 0.203 | 0.797 |
| IL-17D | Pro-inflammatory | 0.203 | 0.544 | 0.263 | 0.797 |
| IL-17E | Pro-inflammatory | 0.507 | 0.751 | 0.977 | 0.999 |
| IL-17F | Pro-inflammatory | 0.735 | 0.878 | 0.682 | 0.971 |
| IL-18 | Pro-inflammatory | 0.042 | 0.373 | 0.685 | 0.971 |
| IL-1α | Pro-inflammatory | 0.258 | 0.544 | 0.568 | 0.971 |
| IL-1β | Pro-inflammatory | 0.156 | 0.524 | 0.760 | 0.971 |
| IL-1 receptor agonist | Anti-inflammatory | 0.038 | 0.373 | 0.389 | 0.812 |
| IL-2 | Pro-inflammatory | 0.230 | 0.544 | 0.002 | 0.107 |
| IL-21 | Other cytokines | 0.537 | 0.751 | 0.347 | 0.797 |
| IL-22 | Other cytokines | 0.508 | 0.751 | 0.733 | 0.971 |
| IL-23 | Other cytokines | 0.482 | 0.751 | 0.741 | 0.971 |
| IL-27 | Other cytokines | 0.281 | 0.570 | 0.580 | 0.971 |
| IL-29/IFNλ1 | Other cytokines | 0.259 | 0.544 | 0.787 | 0.971 |
| IL-2 receptor α | Pro-inflammatory | 0.723 | 0.878 | 0.781 | 0.971 |
| IL-3 | Other cytokines | 0.769 | 0.881 | 0.109 | 0.797 |
| IL-31 | Other cytokines | 0.020 | 0.351 | 0.728 | 0.971 |
| IL-33 | Other cytokines | 0.091 | 0.495 | 0.645 | 0.971 |
| IL-4 | Anti-inflammatory | 0.433 | 0.715 | 0.488 | 0.936 |
| IL-5 | Other cytokines | 0.089 | 0.495 | 0.020 | 0.693 |
| IL-6 | Pro-inflammatory | 0.057 | 0.451 | 0.319 | 0.797 |
| IL-7 | Other cytokines | 0.163 | 0.524 | 0.999 | 0.999 |
| IL-8 | Other cytokines | 0.029 | 0.373 | 0.759 | 0.971 |
| IL-9 | Other cytokines | 0.921 | 0.947 | 0.217 | 0.797 |
| IP-10 | Chemokines | 0.082 | 0.495 | 0.765 | 0.971 |
| I-TAC | Chemokines | 0.358 | 0.652 | 0.837 | 0.971 |
| MCP-1 | Chemokines | 0.185 | 0.524 | 0.720 | 0.971 |
| MCP-2 | Chemokines | 0.755 | 0.878 | 0.284 | 0.797 |
| MCP-3 | Chemokines | 0.077 | 0.495 | 0.306 | 0.797 |
| MCP-4 | Chemokines | 0.634 | 0.818 | 0.343 | 0.797 |
| M-CSF | Other cytokines | 0.260 | 0.544 | 0.840 | 0.971 |
| MDC | Chemokines | 0.216 | 0.544 | 0.186 | 0.797 |
| MIF | Pro-inflammatory | 0.544 | 0.751 | 0.311 | 0.797 |
| MIP-1α | Chemokines | 0.311 | 0.597 | 0.927 | 0.997 |
| MIP-1β | Chemokines | 0.879 | 0.918 | 0.055 | 0.797 |
| MIP-3α | Chemokines | 0.210 | 0.544 | 0.073 | 0.797 |
| MIP-3β | Chemokines | 0.402 | 0.696 | 0.836 | 0.971 |
| MIP-5 | Chemokines | 0.497 | 0.751 | 0.279 | 0.797 |
| SDF-1α | Chemokines | 0.033 | 0.373 | 0.367 | 0.797 |
| TARC | Chemokines | 0.494 | 0.751 | 0.522 | 0.949 |
| TNFα | Pro-inflammatory | 0.161 | 0.524 | 0.136 | 0.797 |
| TNFβ | Other cytokines | 0.142 | 0.524 | 0.991 | 0.999 |
| TPO | Other cytokines | 0.862 | 0.918 | 0.285 | 0.797 |
| TRAIL | Other cytokines | 0.239 | 0.544 | 0.064 | 0.797 |
| TSLP | Other cytokines | 0.972 | 0.979 | 0.245 | 0.797 |
| VEGF | Pro-inflammatory | 0.742 | 0.878 | 0.098 | 0.797 |
| YKL-40 | Other cytokines | 0.184 | 0.524 | 0.793 | 0.971 |

^a^Assessed by separate Cox regression based on baseline concentration level (PFS ~ Concentration level + Treatment + Concentration level: Treatment) by cytokine and treatment in patients with iNHL.
^b^Assessed by separate Cox regression based on baseline concentration level (OS ~ Concentration level + Treatment + Concentration level: Treatment) by cytokine and treatment in patients with iNHL.
CTACK, cutaneous lymphocyte-associated antigen; ENA, epithelial neutrophil-activating protein; EPO, erythropoietin; FLT3L, FMS-like tyrosine kinase 3 ligand; G-CSF, granulocyte colony-stimulating factor; GM-CSF, granulocyte macrophage colony-stimulating factor; GRO, growth-regulated oncogene; IP, IFNγ-inducible protein; I-TAC, IFN-inducible T-cell α chemoattractant; MCP, monocyte chemoattractant protein; M-CSF, macrophage colony-stimulating factor; MDC, macrophage-derived chemokine; MIF, macrophage migration inhibitory factor; MIP, macrophage inflammatory protein; SDF, stromal cell-derived factor; TARC, thymus- and activation-regulated chemokine; TPO, thrombopoietin; TRAIL, TNF-related apoptosis-inducing ligand; TSLP, thymic stromal lymphopoietin; YKL, tyrosine lysine leucine.

**Supplementary Table S5.** Results from cross-validated penalized Cox regression analysis of the association between PFS and mutation status and clinical variables in the C+R arm.

| Variable | Level vs. reference level | HR [95% CI]^a^ | p-value |
| --- | --- | --- | --- |
| In C+R: IL-2 | Low concentration vs. high concentration | 0.334 [0.179-0.625] | 0.0006 |
| In P+R: IL-2 | Low concentration vs. high concentration | 1.730 [0.838-3.571] | 0.1384 |
| In IL-2 low concentration | C+R vs. P+R | 0.358 [0.179-0.717] | 0.0038 |
| In IL-2 high concentration | C+R vs. P+R | 1.852 [0.980-3.500] | 0.0577 |
| ECOG | ECOG = 0 vs. ECOG ≥ 1 | 0.492 [0.314-0.770] | 0.0019 |
| Bulky disease | No vs. Yes | 0.450 [0.233-0.869] | 0.0174 |
| Tumor size | - | 1.004 [0.998-1.010] | 0.1527 |

^a^Assessed by multivariate Cox regression based on baseline concentration level (OS ~ Concentration level + Treatment + Concentration level: Treatment + ECOG + Bulky disease + Tumor size) in patients with iNHL. The covariates were selected as the top three clinical variables associated with OS as indicated by separate Cox regression by clinical variable.
C, copanlisib; P, placebo; R, rituximab

**References**

1. Matasar MJ, Capra M, Özcan M, Lv F, Li W, Yañez E, et al. Copanlisib plus rituximab versus placebo plus rituximab in patients with relapsed indolent non-Hodgkin lymphoma (CHRONOS-3): a double-blind, randomised, placebo-controlled, phase 3 trial. Lancet Oncol 2021;22:678-89.

2. Mafra A, Laversanne M, Gospodarowicz M, Klinger P, De Paula Silva N, Piñeros M, et al. Global patterns of non-Hodgkin lymphoma in 2020. Int J Cancer 2022;151:1474-81.

3. Thandra KC, Barsouk A, Saginala K, Padala SA, Barsouk A, Rawla P. Epidemiology of non-Hodgkin's lymphoma. Med Sci 2021;9:5.

4. National Cancer Institute. SEER Incidence Data, November 2022 Submission (1975-2020), SEER 22 registries. Available at: <https://seer.cancer.gov/statistics-network/explorer/>. Accessed November 10, 2023.

5. Casey M, Odhiambo L, Aggarwal N, Shoukier M, Islam KM, Cortes J. Representation of the population in need for pivotal clinical trials in lymphomas. Blood 2023;142:846-55.
